# Exosomes for Aesthetic Dermatology: A Comprehensive Literature Review and Update

**DOI:** 10.1111/jocd.16766

**Published:** 2025-01-07

**Authors:** Milaan Shah, Victoria Dukharan, Luke Broughton, Carol Stegura, Nina Schur, Luna Samman, Carlos Garcia‐Meraz, Jona Louise D. Macaraeg‐Jimenez, Victoria G. Belo, Lidia Majewska, Ruben‐Gabriel Resendiz Valle, Guillermo Ricardo Vilchis Palacios, Jennifer Roslyn S. De Leon, Todd Schlesinger

**Affiliations:** ^1^ Department of Dermatology Medical University of South Carolina Charleston South Carolina USA; ^2^ Department of Dermatology Kansas City University – GME Consortium/Advanced Dermatology and Cosmetic Surgery Orlando Florida USA; ^3^ School of Medicine Medical University of South Carolina Charleston South Carolina USA; ^4^ Lake Erie College of Osteopathic Medicine Bradenton Florida USA; ^5^ Department of Dermatology Garnet Health Medical Center Middletown New York USA; ^6^ Instituto Panamericano de Profesionales Científicos – Sociedad Mexicana Científica de Medicina Estética Mexico City Mexico; ^7^ Belo Medical Group Philippines; ^8^ ESME Clinic Kraków Poland; ^9^ Apolo Medic Aesthetic Medicine Mexico City Mexico; ^10^ President Medical Group Dexytex Mexico City Mexico; ^11^ Clinical Research Center of the Carolinas Charleston South Carolina USA

**Keywords:** aesthetics, alopecia, exosomes, facial rejuvenation, hair rejuvenation, hyperpigmentation, scar remodeling, scarring, stem cells

## Abstract

**Background:**

Exosomes are nanoscale vesicles derived from various cell types and tissues that have many potential applications, generating great interest from researchers. One particularly intriguing application of exosomes is their use as a direct therapeutic for aesthetic indications. Several studies and case reports have explored the impact of exosomes for numerous cosmetic concerns but a consensus on the outcomes of these studies has not been established.

**Aims:**

In this review, we summarize the proposed mechanism of action, application, and efficacy of treatments with exosomes for alopecia and hair rejuvenation, facial rejuvenation, hyperpigmentation, and scarring.

**Methods:**

We conducted a comprehensive literature review on the use of exosomes for the treatment of alopecia and hair rejuvenation, facial rejuvenation, hyperpigmentation, and scarring. Additionally, several practical clinical cases where exosomes were applied for these indications were included.

**Results:**

The general consensus from the literature review showed that the early evidence supports the efficacy of exosomes for the treatment of alopecia, facial rejuvenation, hyperpigmentation, and scarring. The clinical cases included demonstrated promising improvements in the patients that received treatment. Several limitations regarding the lack of standardization in the production and application of exosomes may limit their current use until more studies are conducted.

**Conclusions:**

Exosomes may serve as a potentially beneficial therapeutic option for several aesthetic dermatologic indications but further investigation is required to fully characterize the scope of their application.

## 1 Introduction

Exosomes refer to membranous, extracellular vesicles, between 30 and 150 nm in caliber, that are capable of exchanging and delivering cellular cargo, including proteins, lipids, RNA, and DNA, between cells [1]. Exosomes are prevalent in several different types of cells, including immune cells, platelets, and stem cells, and are found in numerous biological fluids, such as amniotic fluid, cerebrospinal fluid, breast milk, and serum [1]. Their prevalence in the body and involvement in numerous pathophysiologic processes gives them multiple potential applications and have made them the subject of great interest and investigation by researchers across several medical specialties [1, 2]. This has been exemplified by the use of exosomes as biomarkers for identifying and monitoring different types of cancer, their role in targeted drug delivery given their biocompatibility and reduced immunogenicity, and their therapeutic anti‐apoptotic and anti‐inflammatory properties established in cardiovascular disease research [3, 4, 5].

The use of exosomes as a direct therapeutic has been explored in several studies. Numerous in vivo and in vitro studies have shown that stem cell‐derived exosomes may have great regenerative capacity when used as a direct therapeutic treatment [6]. This evidence has led to the investigation of the use of exosomes for aesthetic indications including hair loss and rejuvenation, facial rejuvenation, hyperpigmentation, and scarring. While the preliminary evidence is positive, the full scope of application and potential effects of exosomes for cosmetic concerns is unclear. Thus, we aim to review and describe the current literature and present relevant clinical cases on the aesthetic indications of exosomes. Additionally, written informed consent was collected from all patients in the included cases. The authors confirm that the ethical policies of the journal, as noted on the journal's author guidelines page, have been adhered to. No ethical approval was required as this is a review article with no original research data.

## 2 Exosomes for Alopecia and Hair Rejuvenation

Exosomes have been investigated as a therapeutic agent for use in alopecia with the goal of inhibiting further hair loss while also potentially inducing additional hair growth. Mechanistically, they interact with the hair follicle in a multitude of ways. Exosomes have been shown to carry Wnt proteins on their surface which cause the activation of β‐catenin, a key gene involved in the signaling pathway for hair growth and regeneration [7, 8, 9]. One of β‐catenin's functions is the induction and maintenance of hair in the anagen phase of the hair cycle [9, 10]. This can potentially contribute to the conversion of hairs into the growth phase of the hair cycle, leading to additional or maintained hair growth, a phenomenon which has been demonstrated in mice models [11]. While the full pathophysiological mechanism is not currently understood, the paracrine signaling of exosomes between cells is believed to be a primary mediator of their influence in hair [11].

Additionally, the source of exosomes has been an important subject of research, as exosomes derived from different cell populations possess differing characteristics. Exosomes derived from dermal papillae cells (DPCs) in hair follicles have been shown to successfully induce hair follicle stem cell proliferation and hair growth while also inhibiting hair cell apoptosis in mouse models [9, 12]. Exosomes from adipose‐derived stem cells (ADSCs) have also been shown to positively induce hair regrowth through DPC proliferation secondary to the upregulation of Wnt/β‐catenin, TNF‐α signaling pathways, and vascular endothelial growth factor expression [9]. There are studies examining the derivation of exosomes from dermal fibroblasts, outer root sheath cells, and bone marrow which all had positive results, but exosomes from DPCs, ADSCs, and mesenchymal stem cells (MSCs) appear to be the most heavily studied in vitro and in mice models [9, 11].

There is considerable preclinical evidence demonstrating the impact of exosomes in mice models. In a study on exosomes from ADSCs, 12 nude mice were grafted with dermal and epidermal cells, of which six also received exosomes with grafting and six served as controls. After 3 weeks, the mice who had received exosomes had significantly more regenerated hairs and follicles compared to that seen in the controls (*p* < 0.001) as well as more terminal hairs present on histology [13]. Additional studies have shown the injection of exosomes derived from DPCs as well as MSCs into mice have promoted the conversion of hairs from the telogen phase to anagen through the activation of DPCs [11, 14]. Benefits have also been seen in models mimicking immune‐mediated alopecia where hair‐depilated mice receiving subcutaneous injections of exosomes from ADSCs had better hair growth, increased hair follicles, and thicker derma compared to controls [15].

Studies on the direct clinical impact of exosomes are currently lacking. There are two clinical trials based out of Iran and Pakistan that are currently enrolling patients to investigate the impact of exosomes on alopecia (NCT05658094, NCT06239207). An additional clinical trial on the use of exosomes in men and women with androgenetic alopecia is scheduled to commence soon (NCT06482541). Currently, no consensus on the appropriate source, dosing, preparation technique, or frequency of dosing is established for exosome treatment, but of note, no significant adverse reactions have been reported to date despite the myriad of experimented applications [11]. Two smaller clinical studies on the efficacy of exosomes have been completed. An analysis of 39 patients who received exosomes from ADSCs showed an increase in mean hair density from 121.7 ± 37.2 hairs/cm^2^ to 146.6 ± 39.5 hairs/cm^2^ (*p* < 0.001) and mean hair thickness from 52.6 ± 10.4 μm to 61.4 ± 10.7 μm (*p* < 0.001) after treatment [16].

Another pilot study with 20 patients receiving exosome treatment also showed increased hair density from 105.4 to 122.7 counts/cm^2^ (*p* < 0.001) and increased mean hair thickness from 57.5 to 64.0 mm (*p* < 0.001) after 12 weeks of treatment [17]. While the initial studies are promising, larger scale clinical trials are still needed to confirm the efficacy of exosomes.

**FIGURE 1:**
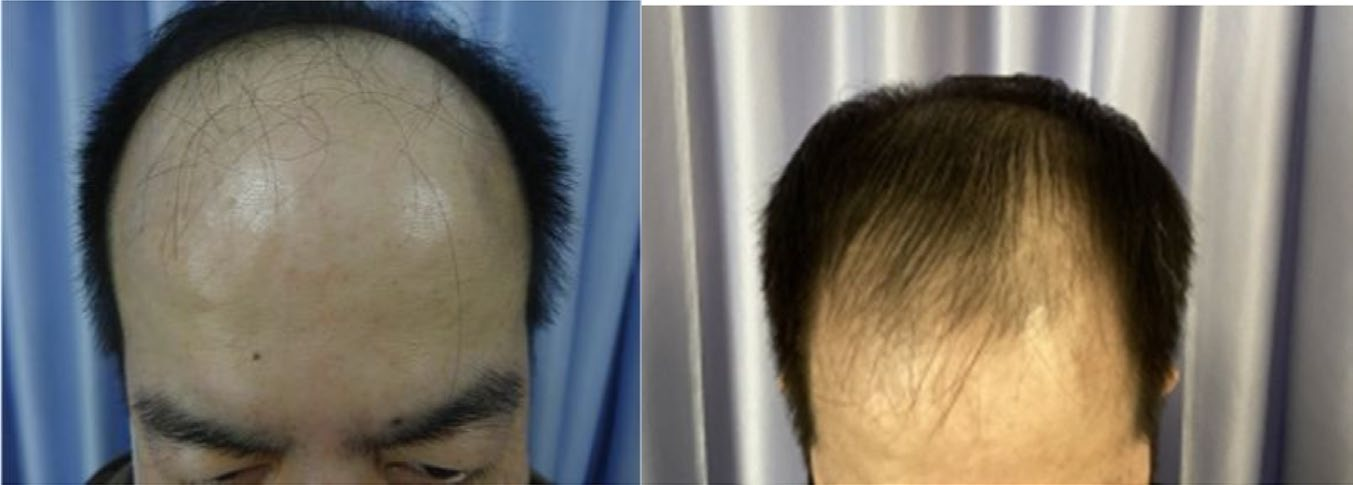
Images of the frontal scalp before and after exosome treatment. Images provided courtesy of Dr. Tomoharu Nakano.

**FIGURE 2:**
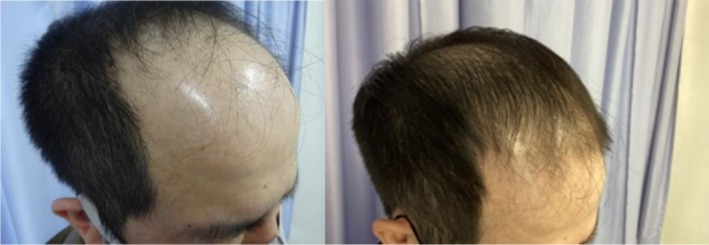
Left frontal view of the scalp before and after exosome treatment. Images provided courtesy of Dr. Tomoharu Nakano.

**FIGURE 3:**
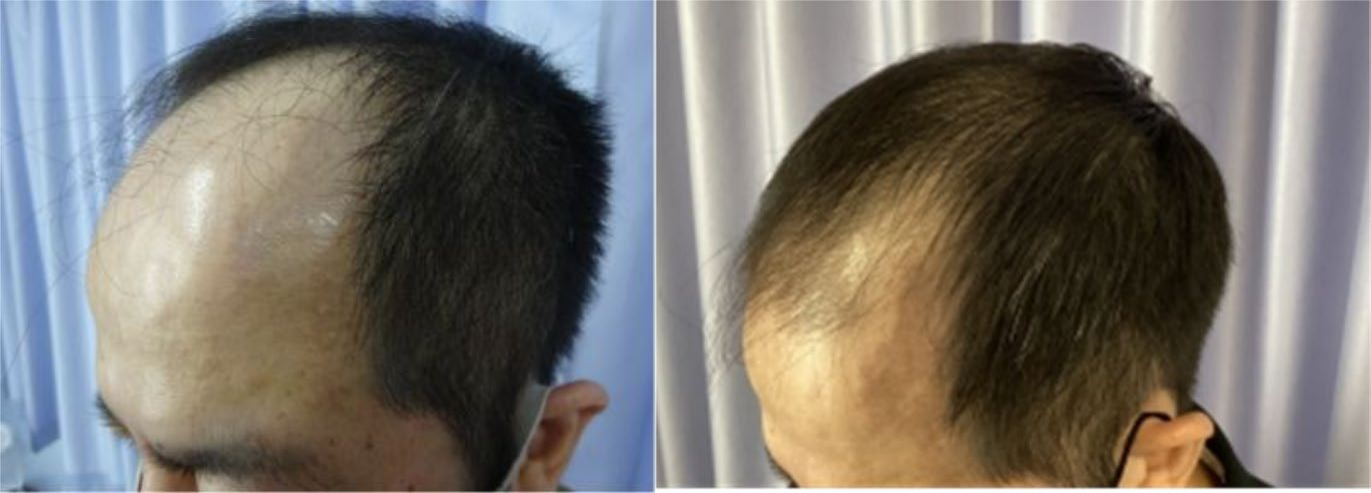
Right frontal view of the scalp before and after exosome treatment. Images provided courtesy of Dr. Tomoharu Nakano.

**FIGURE 4:**
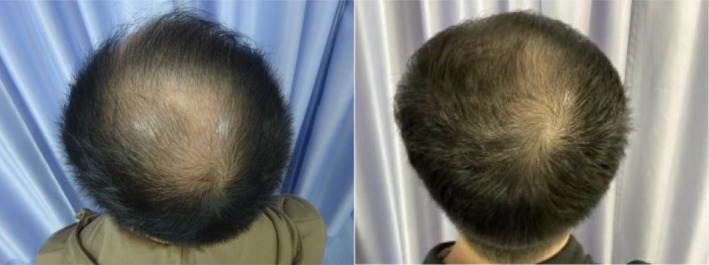
Images of the vertex scalp before and after exosome treatment. Images provided courtesy of Dr. Tomoharu Nakano.

In a recent case, a 48‐year‐old male patient with Norwood 5 stage androgenetic alopecia on a medication regimen of oral dutasteride and topical minoxidil presented for exosome treatment after previously experiencing poor results with ADSCs‐based exosomes. He received five treatments of a plant‐derived exosome complex over the course of 5 months and experienced substantial visible hair growth (Figures 1, 2, 3, 4).

**FIGURE 5:**
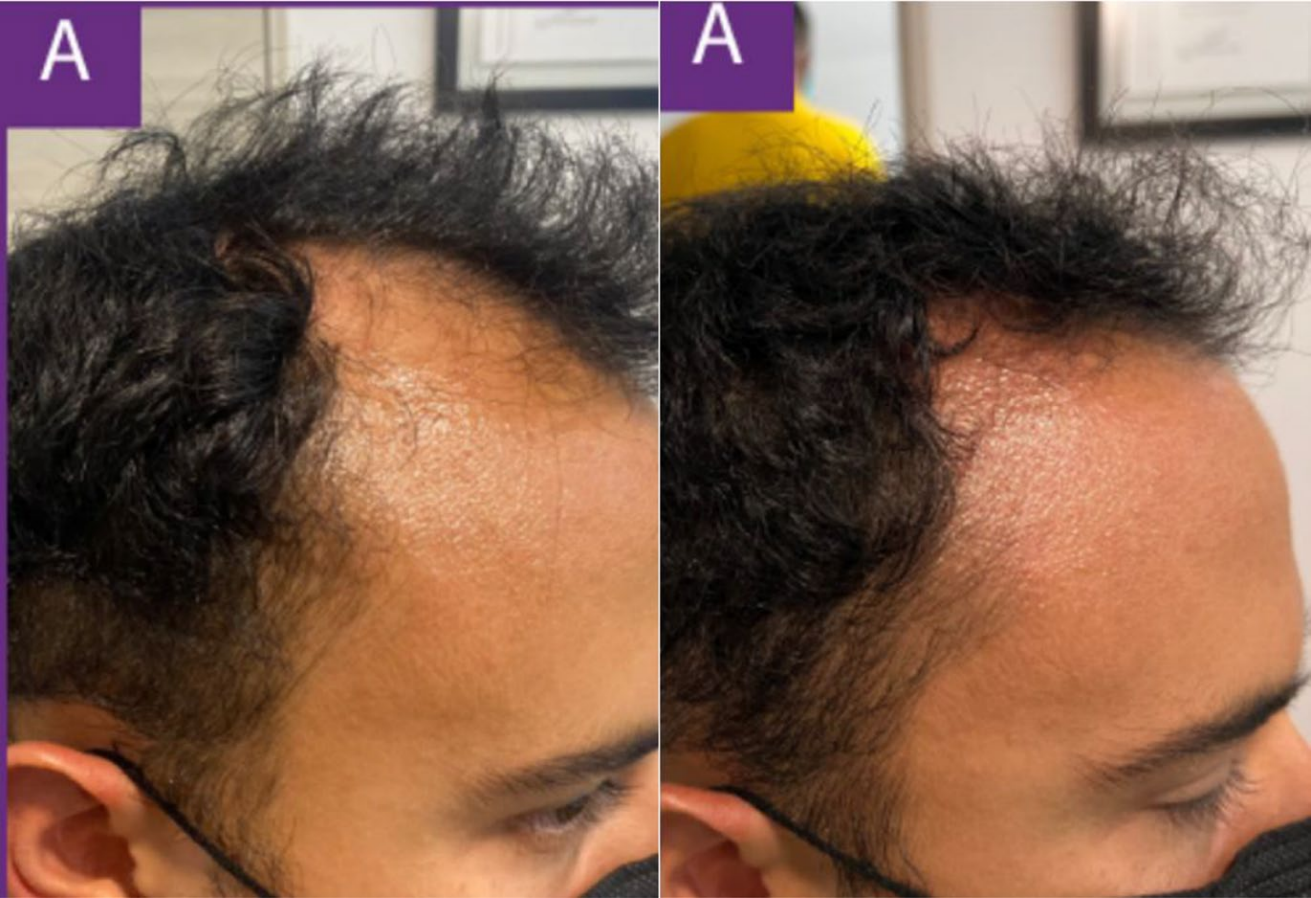
Left frontal view of the scalp before and after exosome treatment. Images provided courtesy of Dr. Carlos Alberto Garcia Meraz.

**FIGURE 6:**
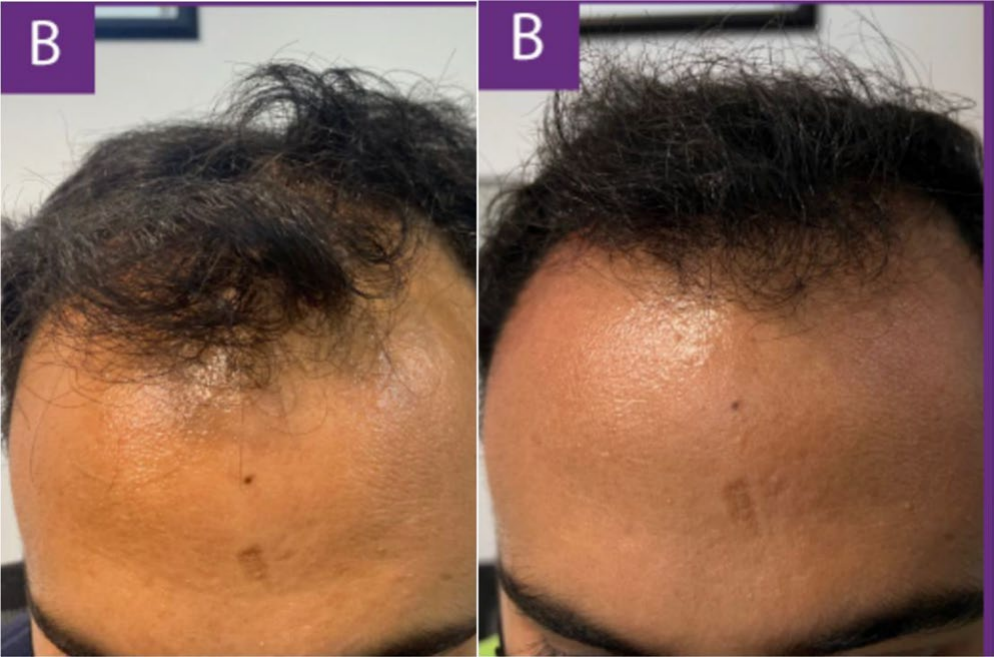
Images of the frontal scalp before and after exosome treatment. Images provided courtesy of Dr. Carlos Alberto Garcia Meraz.

**FIGURE 7:**
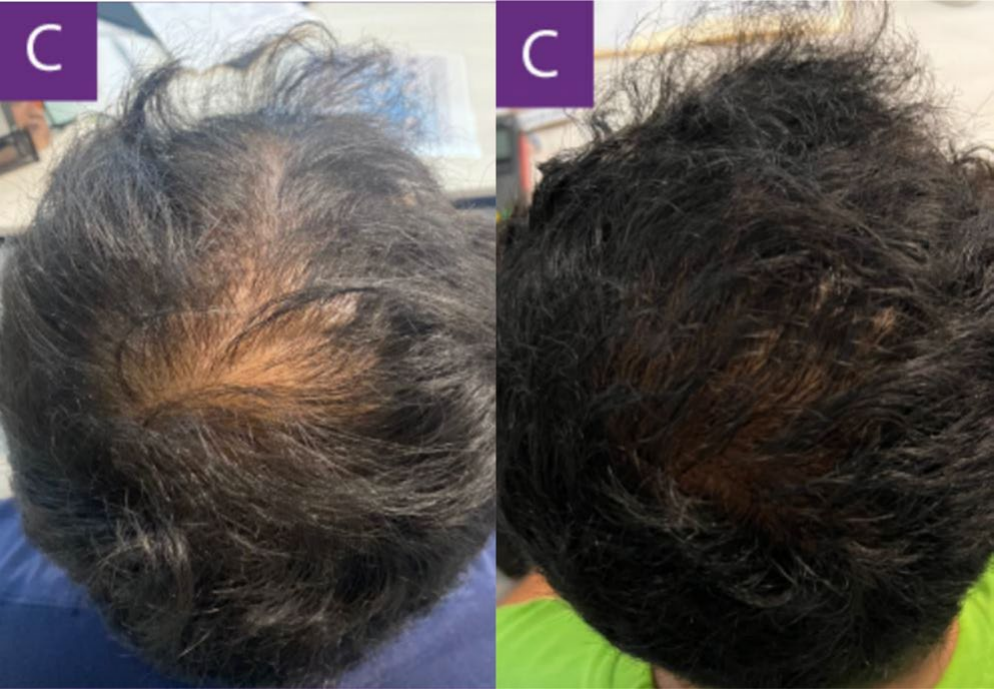
Images of the vertex scalp before and after exosome treatment. Images provided courtesy of Dr. Carlos Alberto Garcia Meraz.

**FIGURE 8:**
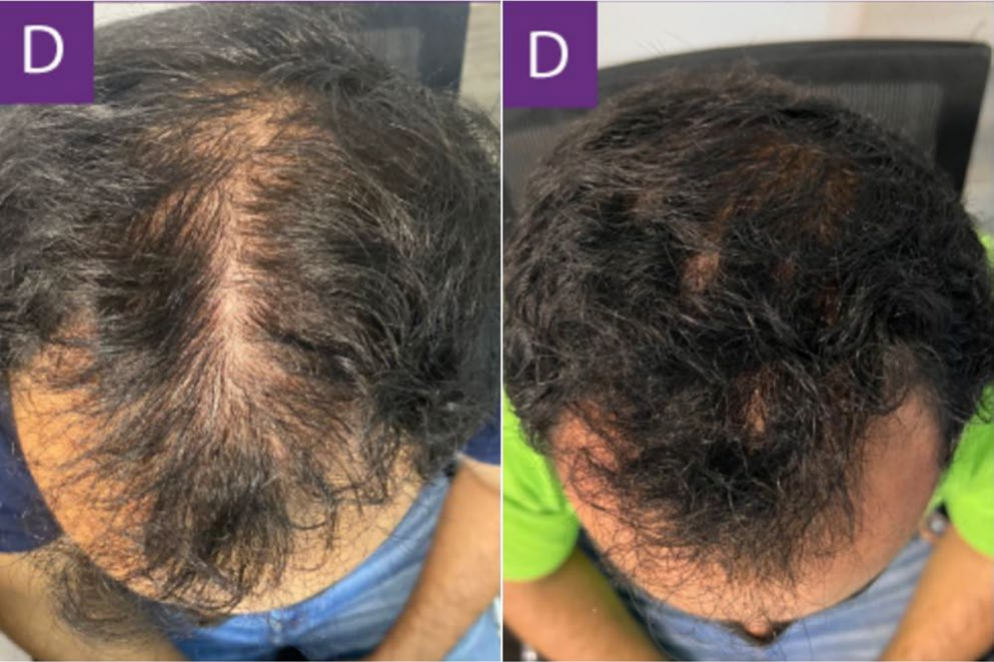
Top view of the scalp before and after exosome treatment. Images provided courtesy of Dr. Carlos Alberto Garcia Meraz.

**FIGURE 9:**
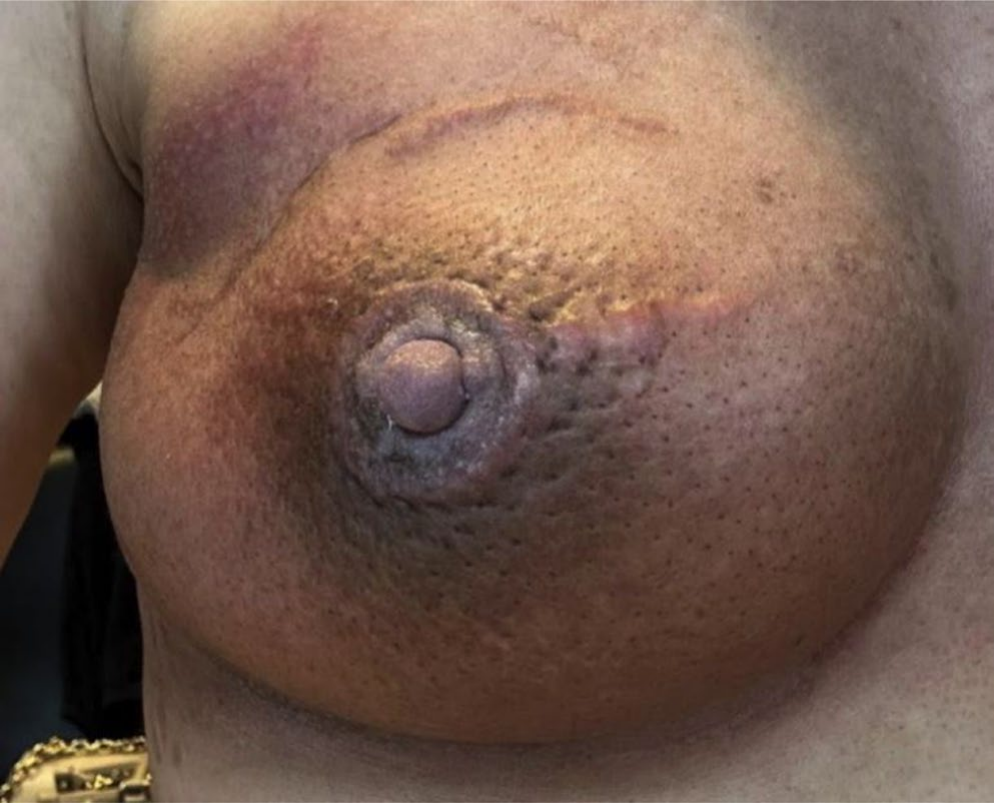
The patient's areola is hyperpigmented and edematous following lumpectomy and radiation therapy to treat invasive breast carcinoma. Images provided courtesy of Dr. Jona Louise Macaraeg‐Jimenez.

**FIGURE 10:**
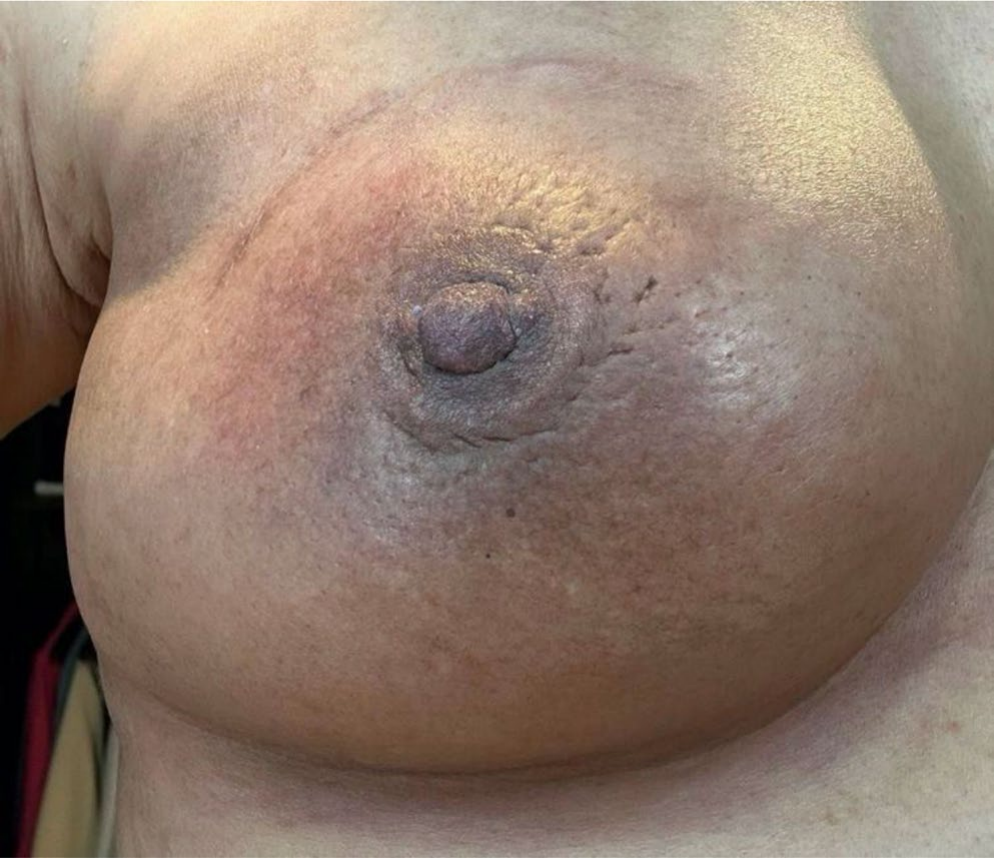
The patient's areola with less hyperpigmentation and edema 2 weeks after completing treatment with twice a day topical exosomes for 3 weeks. Images provided courtesy of Dr. Jona Louise Macaraeg‐Jimenez.

**FIGURE 11:**
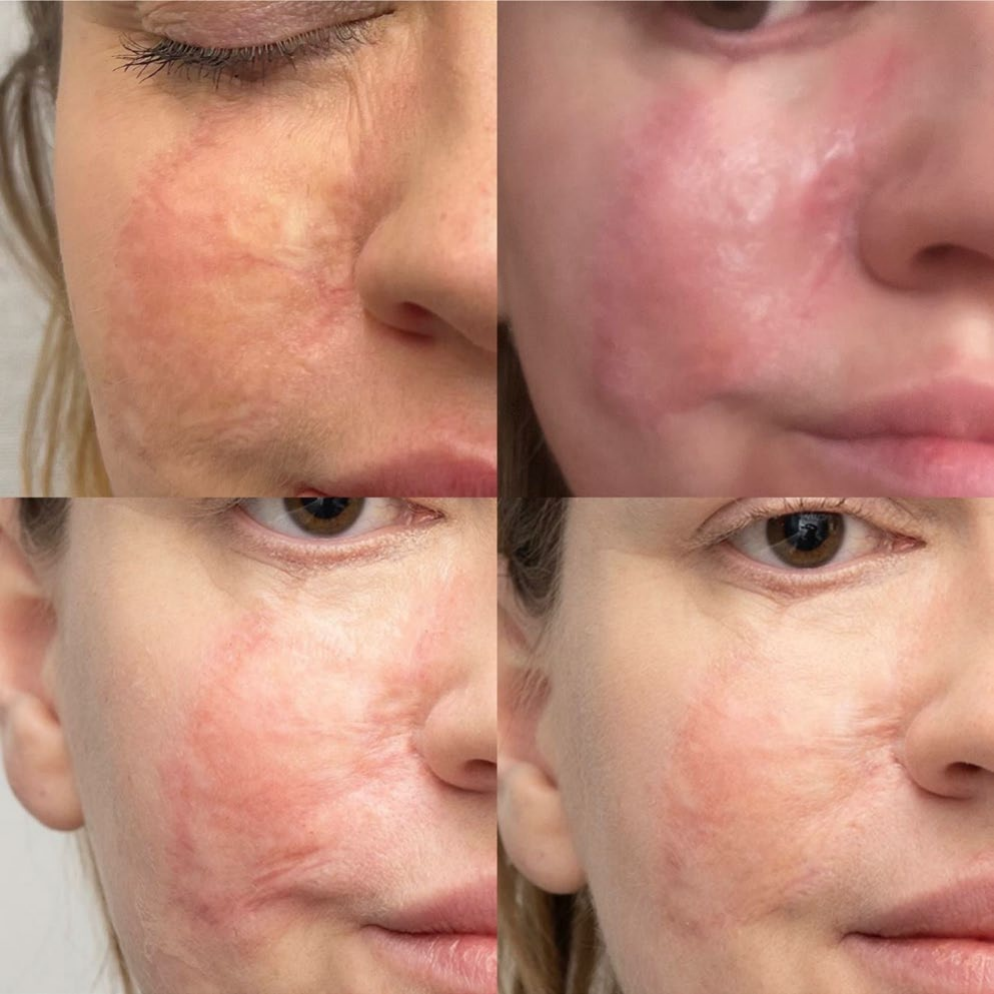
Top Left: Patient's scar before treatment. Top Right: The scar 1 day after the initial treatment. Bottom Left: The scar 10 days after the first treatment. Bottom Right: The patient's scar at day 13 following two microneedling treatments. Images provided courtesy of Dr. Lidia Majewska.

**FIGURE 12:**
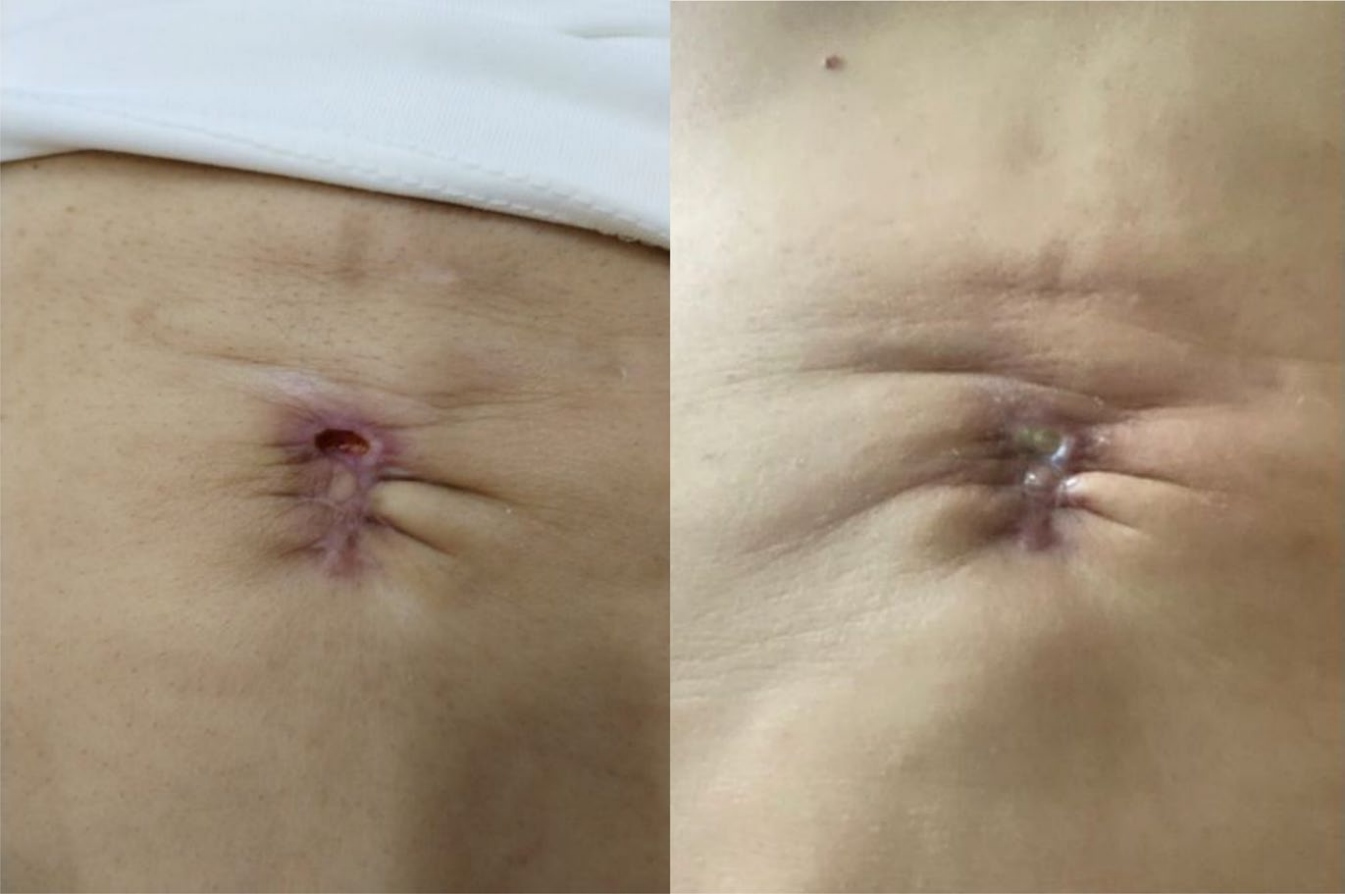
Images of the umbilical scar prior to therapy. Images provided courtesy of Dr. Gabriela Cedillo Garcia.

**FIGURE 13:**
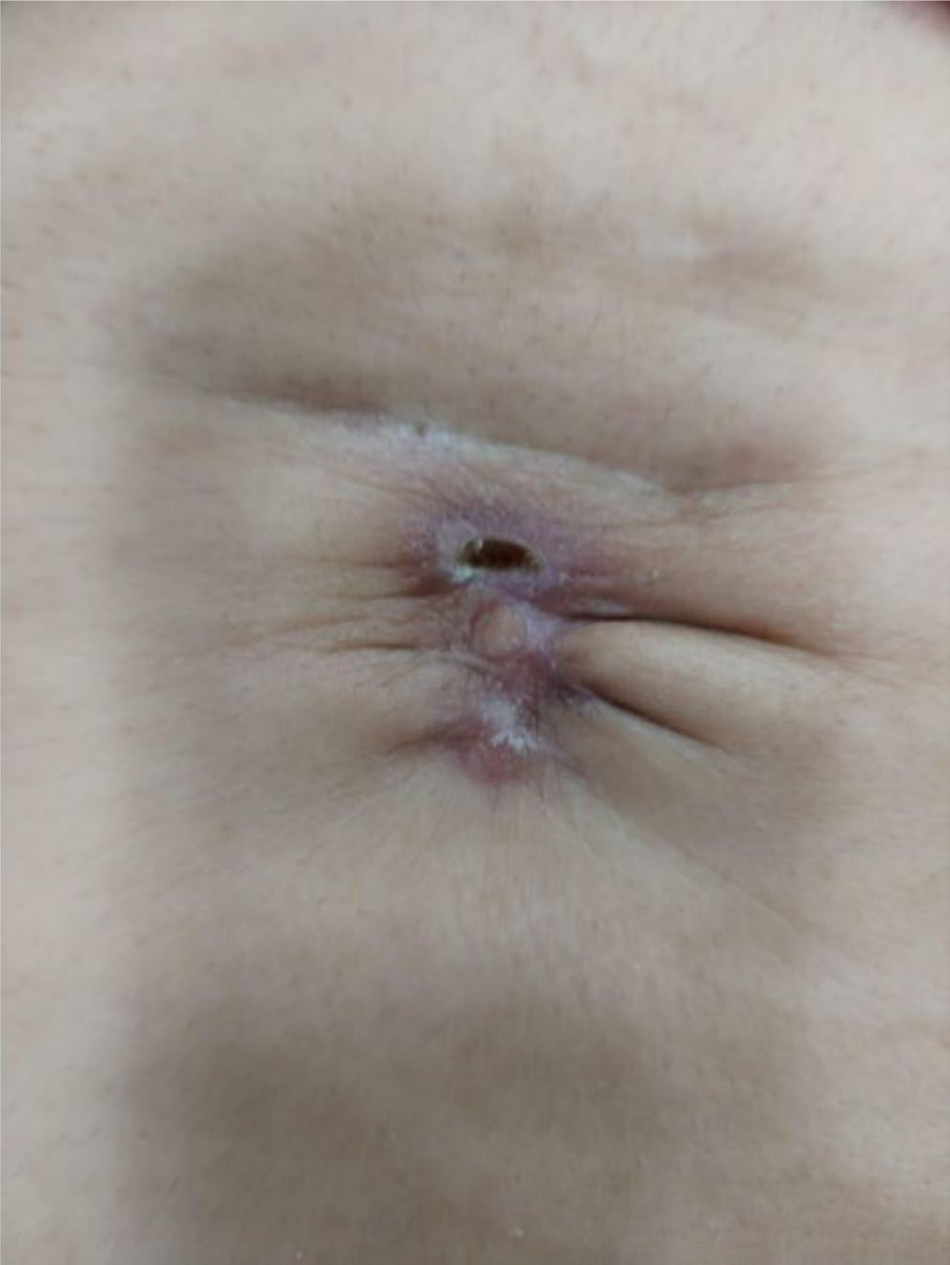
The umbilical scar 8 days after the initial treatment session. Images provided courtesy of Dr. Gabriela Cedillo Garcia.

**FIGURE 14:**
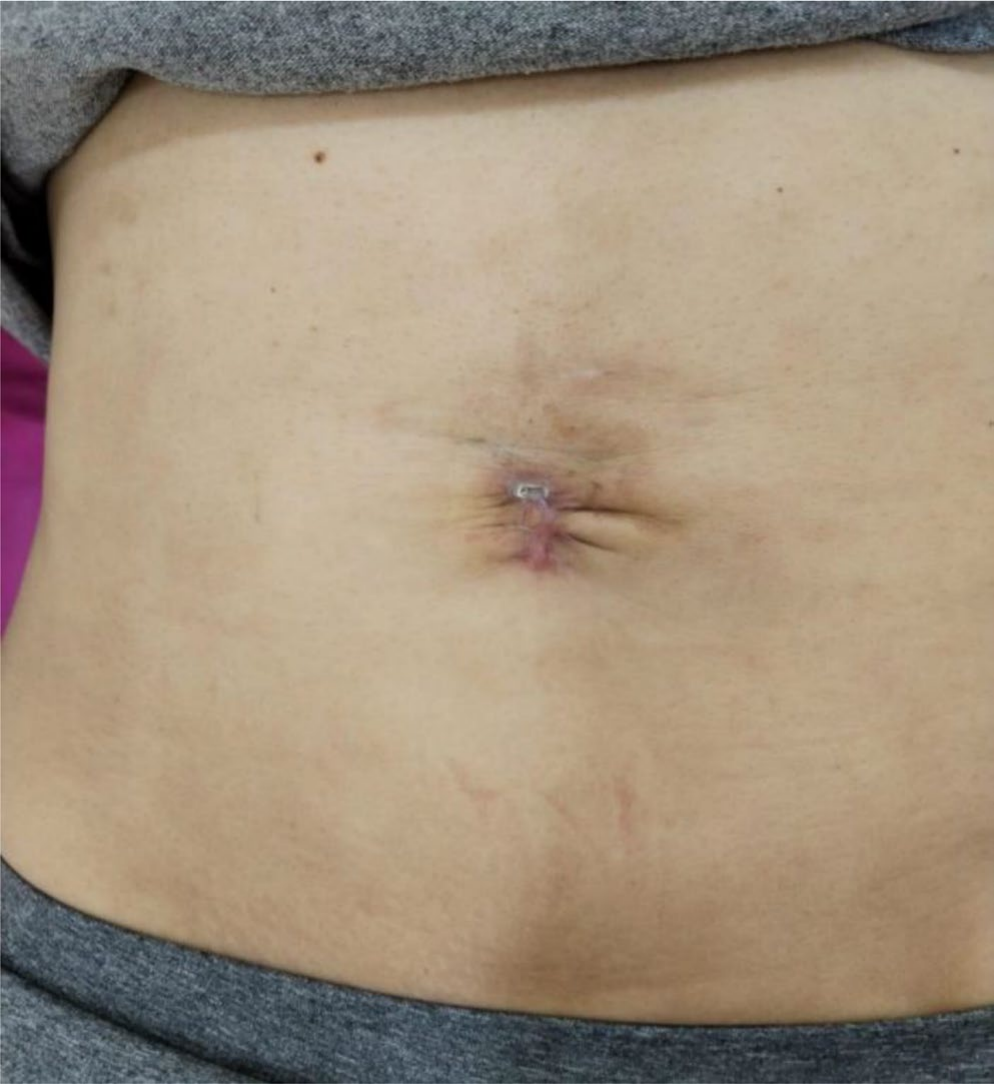
The umbilical scar 7 days after the second treatment session. Images provided courtesy of Dr. Gabriela Cedillo Garcia.

**FIGURE 15:**
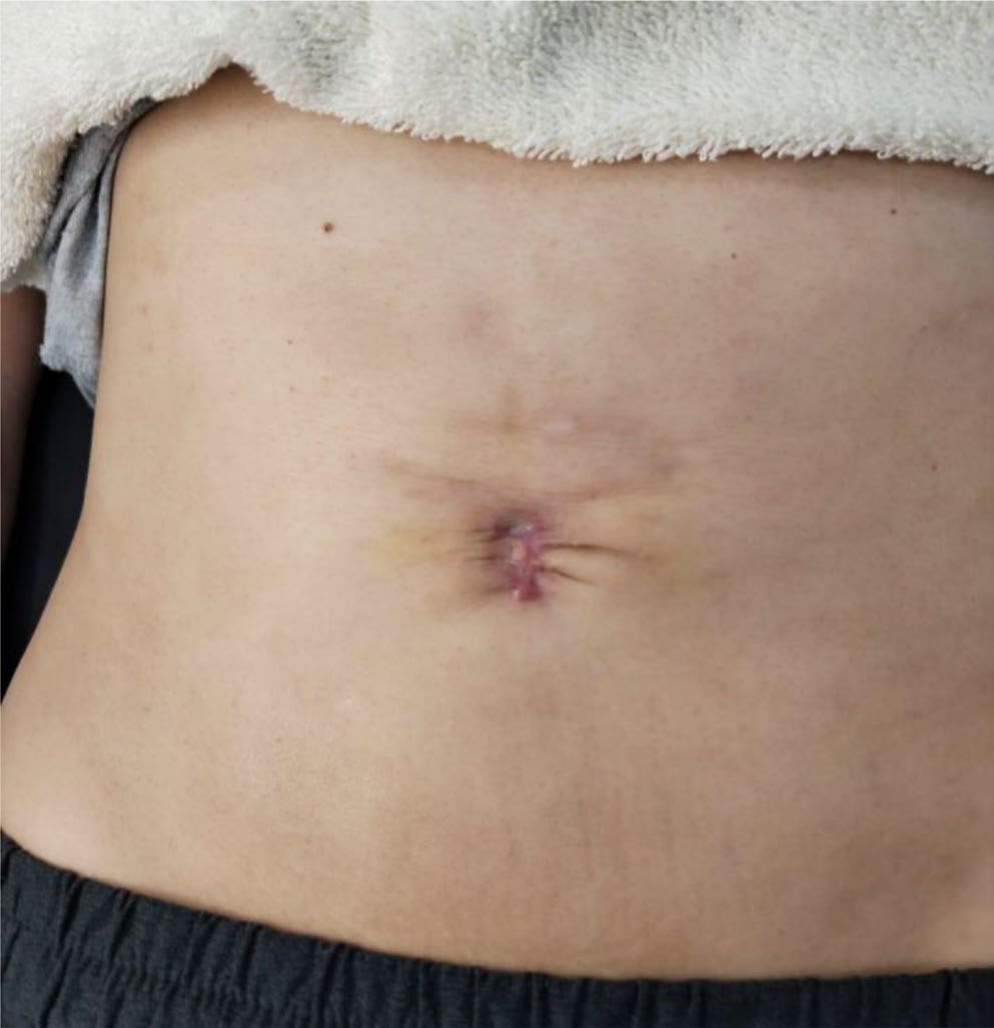
The umbilical scar 7 days after the third treatment session. Images provided courtesy of Dr. Gabriela Cedillo Garcia.

**FIGURE 16:**
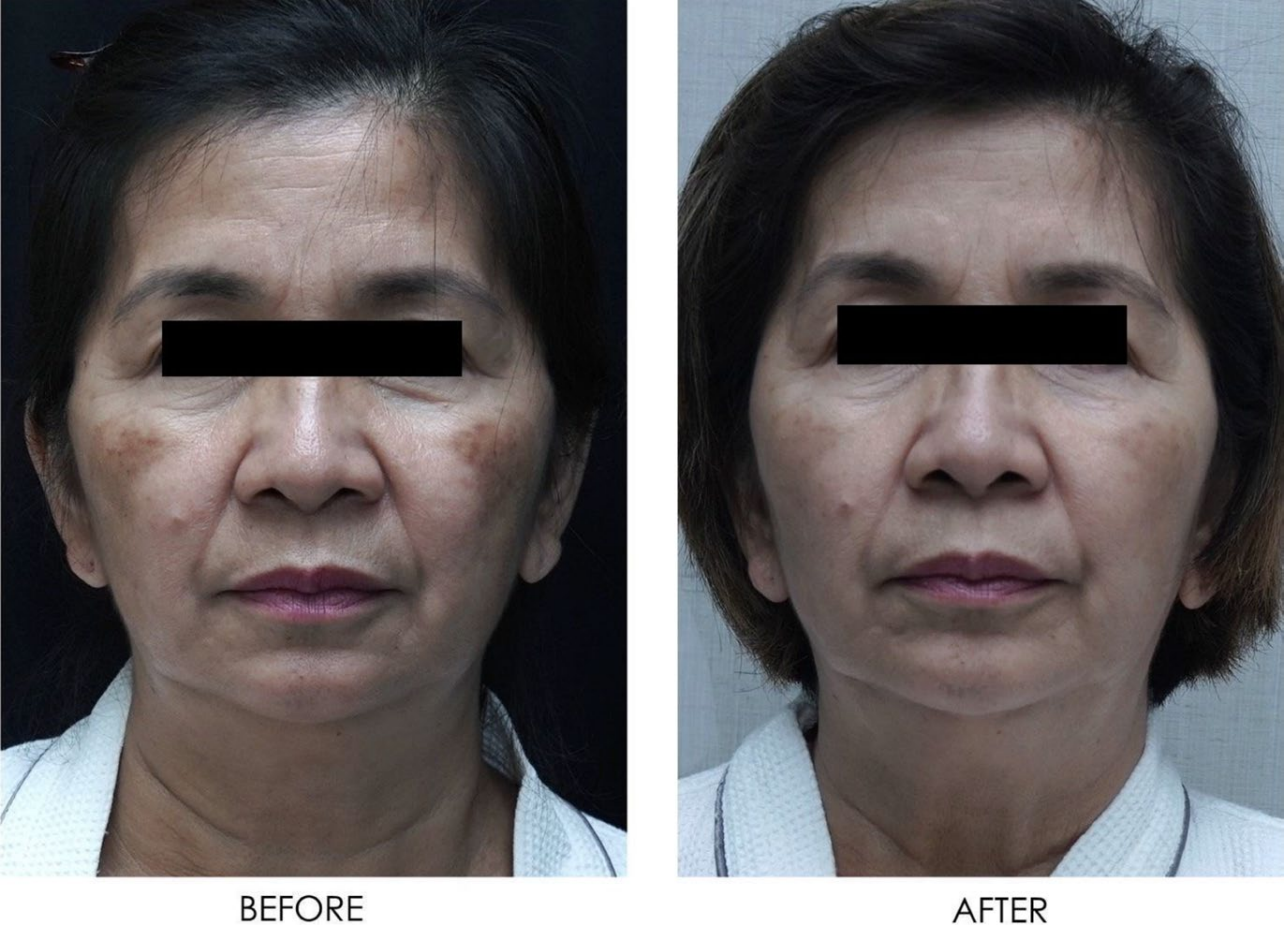
Front‐facing view of the patient before and after exosome treatment. Images courtesy of Dr. Jennifer Roslyn S. De Leon.

**FIGURE 17:**
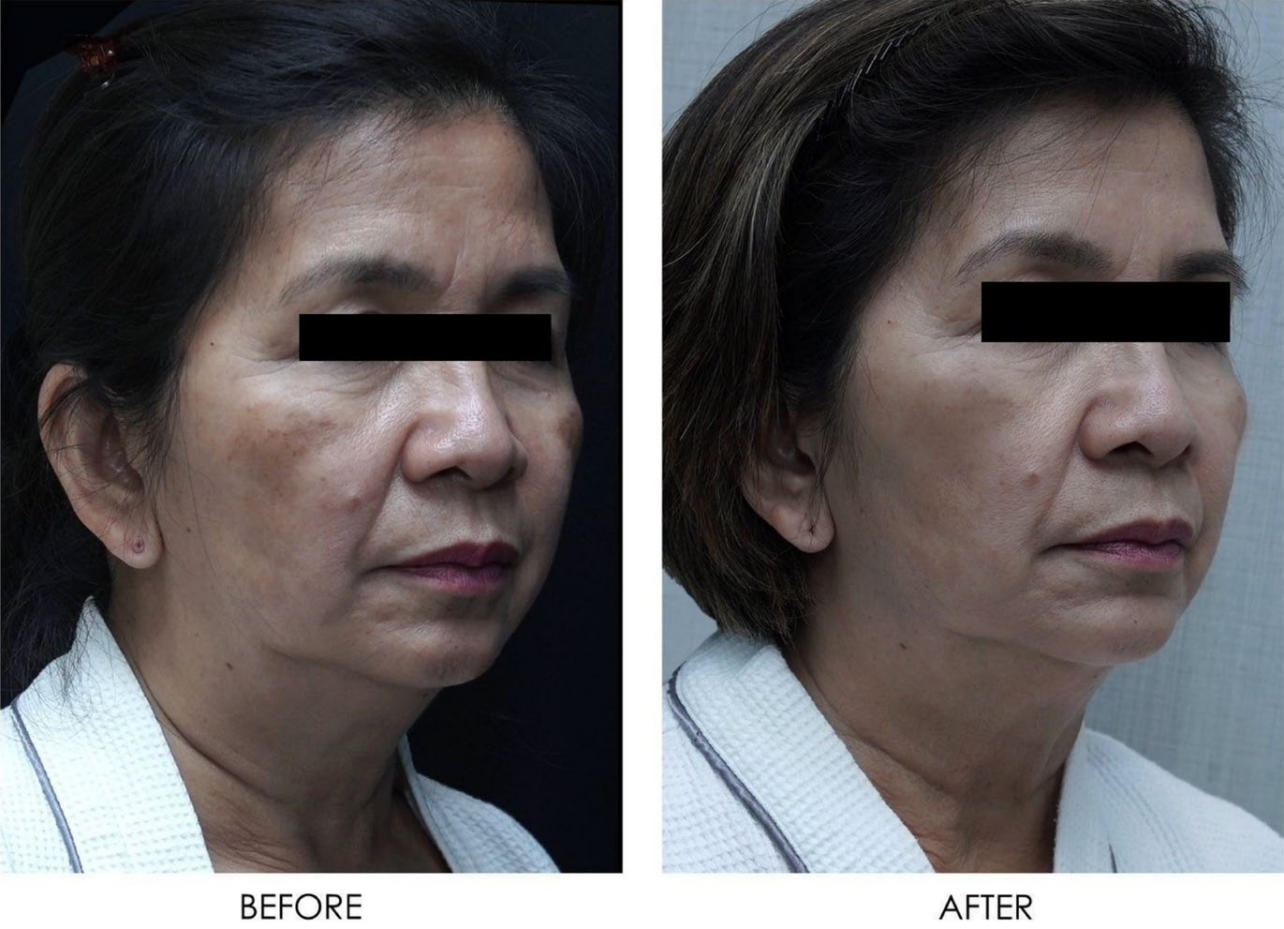
Angled view of the right side of the patient's face before and after exosome treatment, highlighting the cheek area. Images courtesy of Dr. Jennifer Roslyn S. De Leon.

**FIGURE 18:**
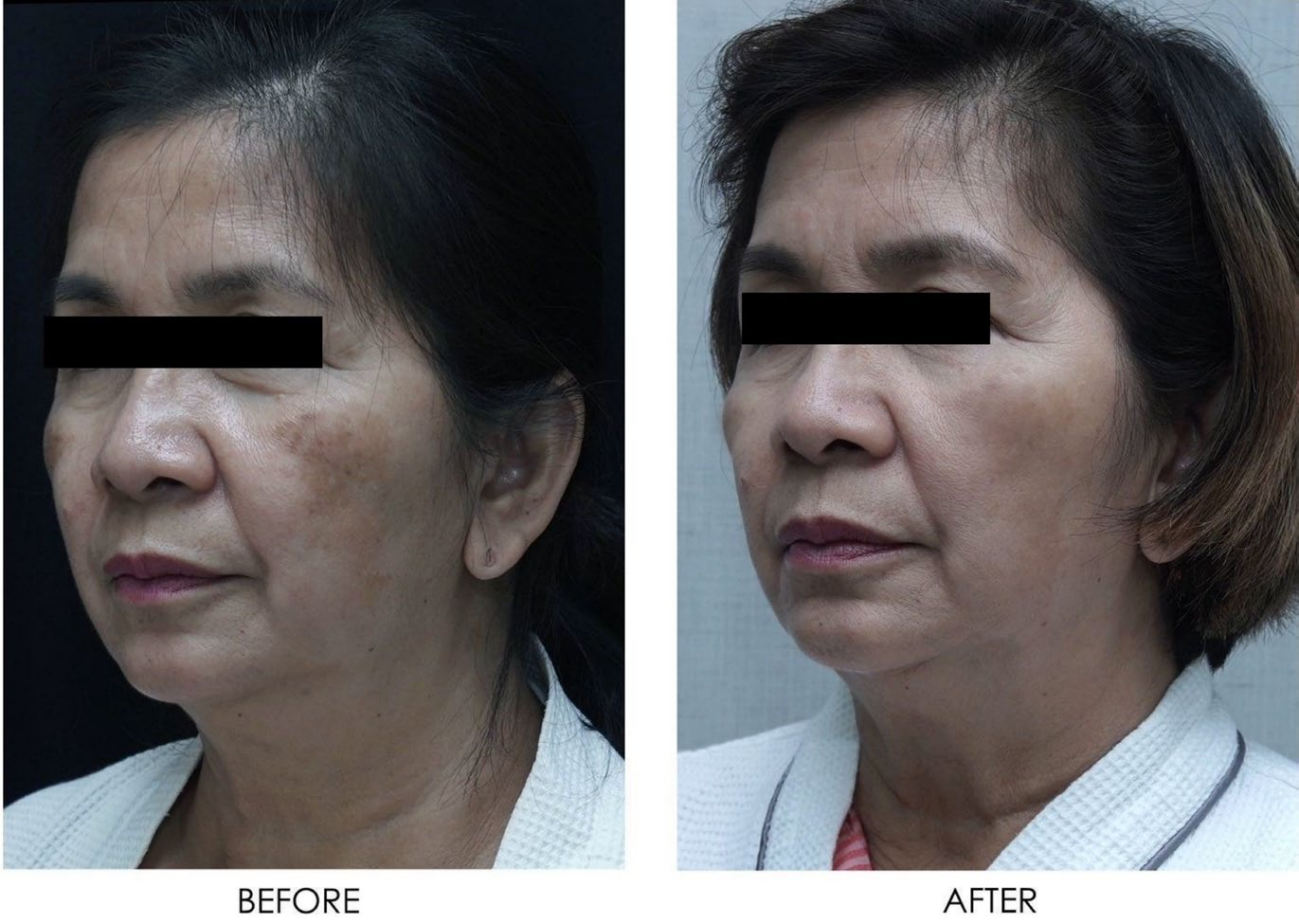
Angled view of the left side of the patient's face before and after exosome treatment, highlighting the cheek area. Images courtesy of Dr. Jennifer Roslyn S. De Leon.

**FIGURE 19:**
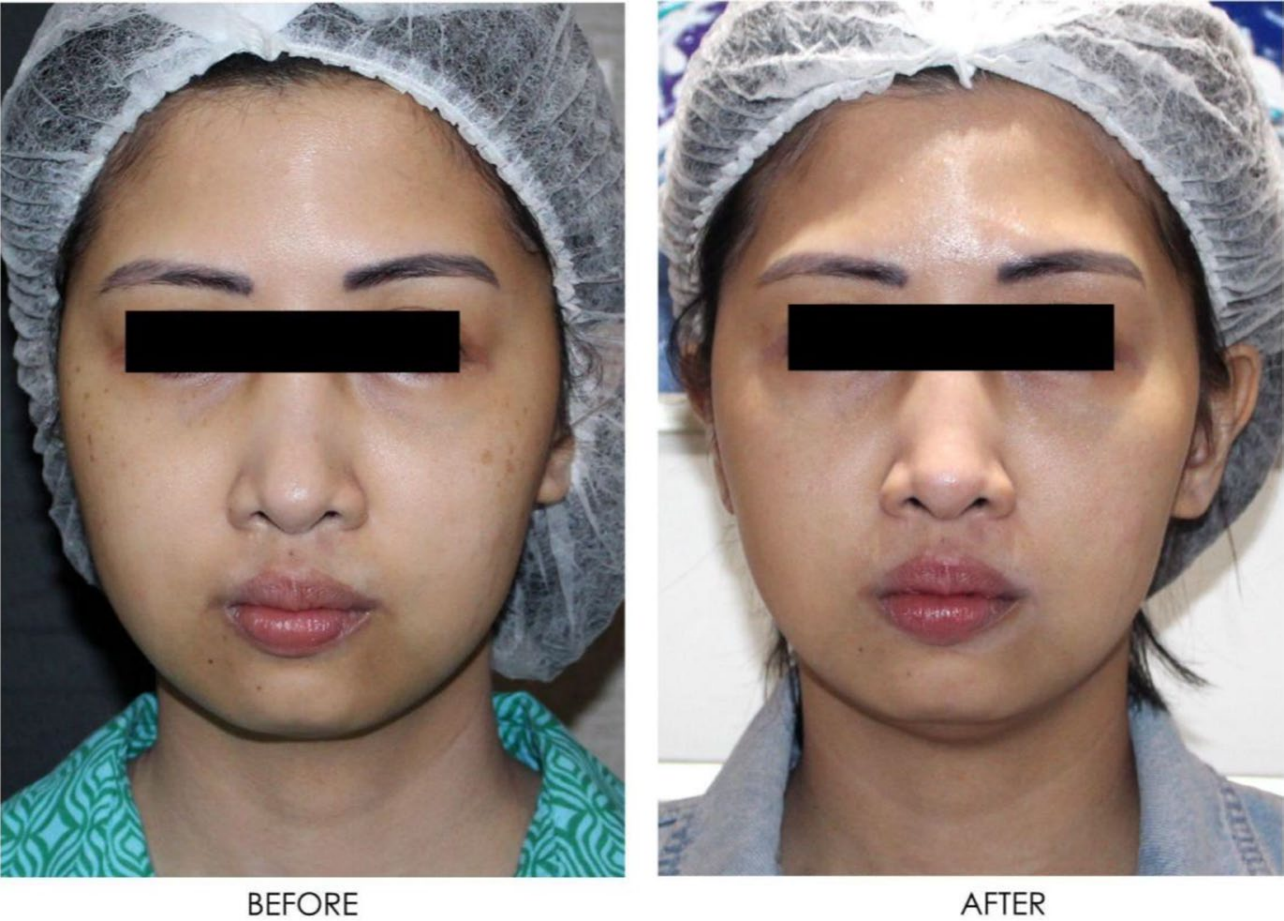
Front‐facing view of the patient before and after exosome treatment. Images courtesy of Dr. Jennifer Roslyn S. De Leon.

**FIGURE 20:**
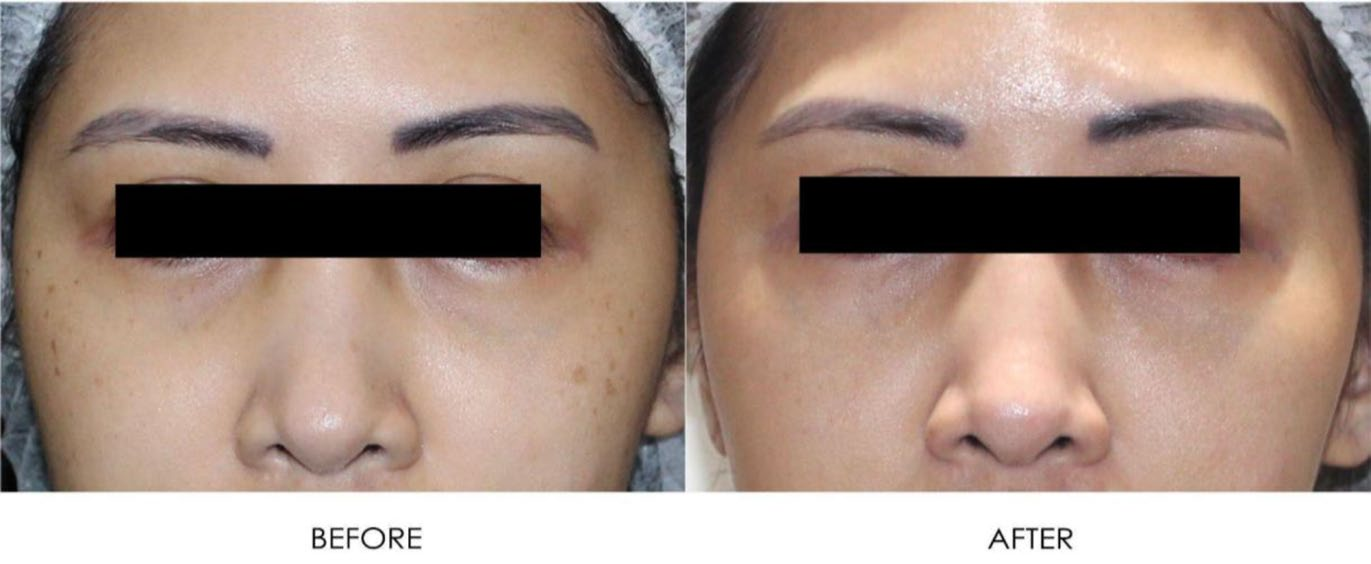
Close up front facing view before and after exosome treatment. Images courtesy of Dr. Jennifer Roslyn S. De Leon.

**FIGURE 21:**
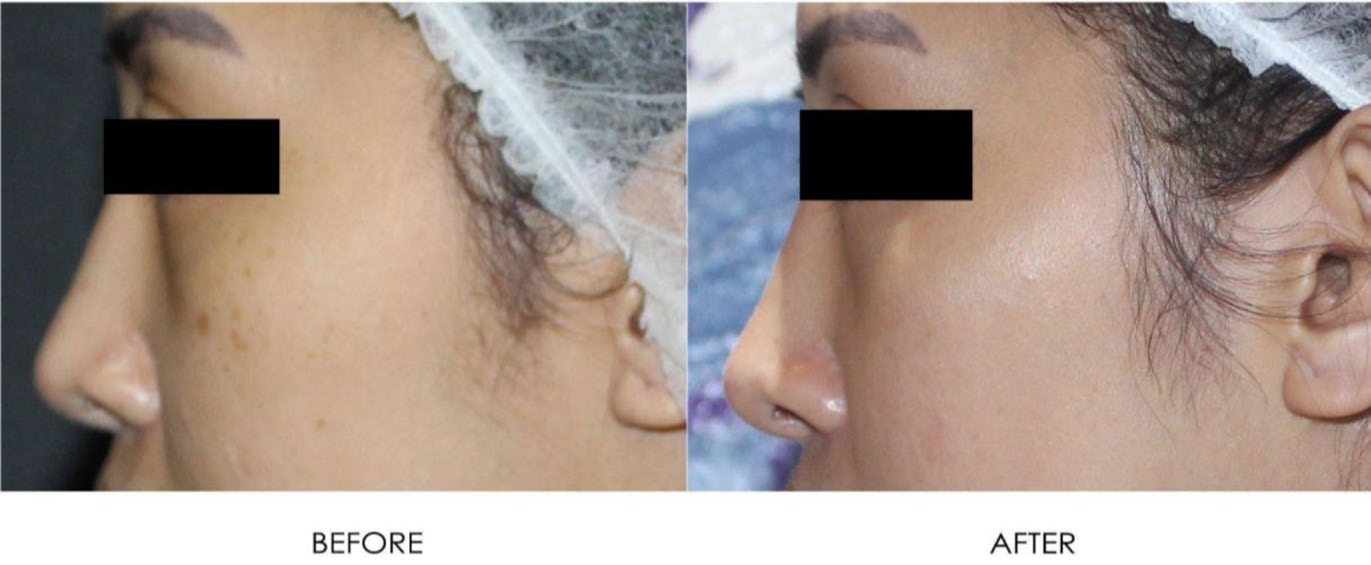
Left side profile before and after exosome treatment. Images courtesy of Dr. Jennifer Roslyn S. De Leon.

**FIGURE 22:**
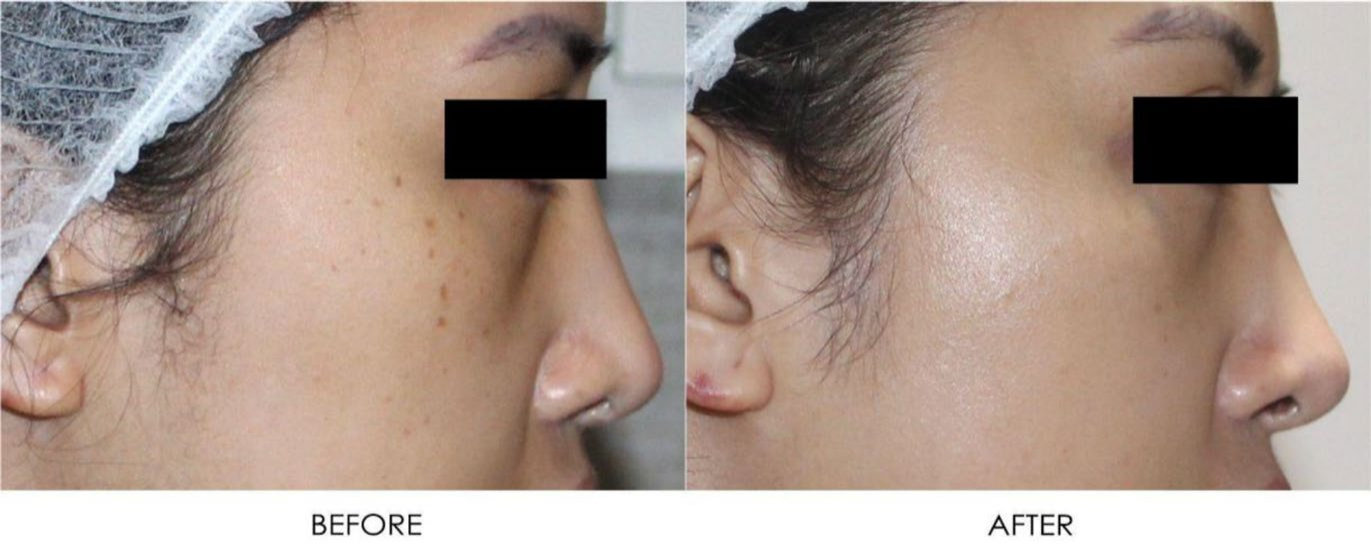
Right side profile view before and after exosome treatment. Images courtesy of Dr. Jennifer Roslyn S. De Leon.

In another case, a 28‐year‐old male with an 8‐year history of alopecia sought consultation for additional treatment options following an acceleration in his shedding over the prior 3 months. The patient had already been on an established regimen of topical minoxidil, 1 mg of oral finasteride daily, and supplementation with several B‐vitamins. The patient was advised to start recurring subcutaneous injections of exosomes derived from human ADSCs every 30 days. [Correction added on July 16, 2025, after first online publication: Interval between treatments was updated.] The following results show the patient 90 days after his initial treatment (Figures 5, 6, 7, 8). [Correction added on July 16, 2025, after first online publication: Follow up time frame was corrected.]

## 3 Exosomes for Scar Remodeling

Exosomes derived from various sources have been investigated for their use with improved scar healing and remodeling given their potential induction of cellular proliferation and immunomodulatory properties. MSCs, in particular, have been found to be beneficial in regulating the immune system response to injury by altering fibroblast activation and promoting angiogenesis [18]. Thus, exosomes derived from MSCs have been studied for their possible benefits in ameliorating and rapidly healing scars.

Using an in vivo mouse model, mice with full thickness wounds were injected with MSC‐derived exosomes modified with tumor necrosis factor‐inducible gene 6 protein (TSG‐6) overexpression to examine their impact on pathological wound healing. Analysis of the tissue post‐injection found several features consistent with decreased scar injury, including decreased inflammatory molecule release and reduced collagen deposition [19]. Rat models with wounds treated in vivo with exosomes derived from human amniotic epithelial cells (hAECs‐Exo) have also demonstrated positive attributes of improved and accelerated wound healing and organized collagen fiber deposition not resulting in scar [20]. hAECs‐Exo are thought to promote wound healing and prevent scarring by causing the proliferation and migration of fibroblasts while also reducing extracellular matrix deposits through the stimulation of matrix metalloproteinase‐1 (MMP‐1) [20].

In regards to keloid and hypertrophic scar treatments through exosomes, ADSCs‐derived exosomes are the most commonly used and studied intervention [21]. ADSC exosomes have been shown to have the potential to inhibit proliferation and extracellular matrix production caused by keloid fibroblasts in the late stages of wound healing [22]. Additionally, there is evidence that ADSC‐derived exosomes can inhibit the TGF‐B1/Smad pathway leading to decreased proliferation, migration, and collagen production of keloid fibroblasts while also inducing the apoptosis of keloid fibroblasts [23]. MSC‐derived and ADSC‐derived exosomes have also been demonstrated to have anti‐fibrotic functions and cause the disruption of angiogenesis in keloid tissue [21]. The preliminary evidence for the use of exosomes for scar prevention and hypertrophic scar/keloid treatment is positive, and further clinical application of these therapies for these indications appears warranted.

In a recent case, a 48‐year‐old female with a history of invasive breast carcinoma treated with lumpectomy and radiation therapy had resulting hyperpigmentation around the nipple. The breast tissue was also erythematous and edematous (Figure 9). For treatment, an exosome balm product was applied to the area twice a day for 3 weeks, with the results being seen at week five, 2 weeks after treatment was completed (Figure 10). The patient had reported significant improvement in hyperpigmentation after 3 weeks of treatment, and there were no adverse effects reported.

In an additional case, topical exosomes were used for scar and skin atrophy treatment. A 36‐year‐old female had received an autologous skin graft to the middle face 30 years ago, which left a visible scar. The patient underwent microneedling followed by topical exosomes application in three layers. The patient then applied topical exosomes twice daily for 10 days. She returned for a second microneedling treatment after 10 days and continued her home regimen. On day 13, the patient reported significant improvements in the margins of the graft. The scar appeared less visible and thinner, and the discoloration had decreased (Figure 11).

Another case displays the use of exosomes for treatment of scarring following wound dehiscence and post‐operative infection of an umbilical scar of a 42 year old female. Exosomes were delivered through microneedling with collagenase enzymes at the hypodermis. The treatment started with an injection of reconstituted collagenase enzymes with saline and lidocaine at the umbilical scar. Punctures were separated by 1 cm. Then, exosomes were distributed over the area and applied with a dermapen to a depth of 0.75 mm for four repetitions. Four sessions were completed in total. The patient rated her satisfaction as a 10/10 for improvement of skin texture, decrease in folds, closure of dehiscence of the umbilical scar, and unification of the placement. Results were documented prior to the procedure (Figure 12), 8 days after the first session (Figure 13), 7 days after the second session (Figure 14), and 7 days after the third session (Figure 15).

## 4 Exosomes for Hyperpigmentation

Hyperpigmentation is the darkening of areas of skin secondary to excess melanin production [24]. Hyperpigmentation is commonly seen in melasma and post‐inflammatory hyperpigmentation and can result from various factors including UV exposure, hormonal changes, and inflammatory response. Traditional treatments include options such as topical agents and chemical peels, but these have limited efficacy and carry potential side effects. Furthermore, treatment of hyperpigmentation generally requires long‐term adherence and high patient compliance to be successful. Thus, there is a need for improved therapies that may yield better results. The initial evidence supports the use of exosomes for the effective treatment of hyperpigmentation.

Exosome therapy is hypothesized to impact pigmentation through their modulation of cytokines and molecules involved in inflammatory responses including inflammatory factor‐interleukin 1 (IF‐1), MMP‐1, MMP‐3, collagen 1 (COLA1), and COLA3 [24]. A study by Cicero et al. found that the exosomes secreted by keratinocytes were engulfed by melanocytes and impacted the production of melanin when the exosomes were treated with UV‐B light [25]. They postulated that this response may be impacted by the type of miRNAs carried by the exosomes. Another study by Kim et al. presented an alternative interaction between exosomes and melanocytes and found that exosomes inhibited the synthesis of melanin through regulation of the microphth‐associated transcription factor (MITF) through activation of the extracellular signal‐regulated kinase (ERK) pathway [26]. Suppression of MITF leading to decreased melanogenesis was also shown by Wang et al. in mouse melanoma cells treated with ADSC‐derived exosomes that carried miR‐181a‐5p and miR‐199a [27].

Another study by Lee et al. showed that proteins required for melanosome transport, such as rab27a and MLPH, were downregulated in mice and melanoma cells treated with exosomes [28]. In an in vitro and clinical study, Cho et al. [29] found that the application of exosomes was effective in treating hyperpigmentation in cells as well as increasing skin brightness in a randomized placebo‐controlled study. In the in vitro hyperpigmentation study, ADSC‐derived exosomes applied in mouse melanoma cells yielded reduced rates of melanin production. Additionally, the anti‐pigmentation effect of the exosomes was present in both the absence and presence of ɑ‐melanocyte stimulating hormone (ɑ‐MSH). They also investigated how exosomal therapy impacts hyperpigmentation in 21 female volunteers. Then, in a split‐face, placebo‐controlled trial with 21 female patients, the administration of exosomes twice daily for 8 weeks demonstrated significant improvements in skin brightening and melanin reduction in the treatment group compared to the controls. These results support the idea that the mechanism behind exosomal therapy for hyperpigmentation is likely a multifactorial process involving several different proteins and other bioactive molecules. The use of exosomes in clinical practice shows promise, but additional research is needed to further elucidate the mechanisms behind the role of exosomes on pigmentation.

Figures 16, 17, 18 demonstrate the before (left) and after (right) images of a patient with melasma who underwent treatment with Picosure Pro and exosomes. The patient rated her satisfaction with the treatment as a 10/10 with lightening of areas of hyperpigmentation on her cheeks noted after treatment.

## 5 Exosomes for Facial Rejuvenation and Anti‐Aging

Skin rejuvenation, including improved rigidity, reduction of wrinkles, and the reversal of other signs of aging of the skin, is an indication of significant interest. Aging of the skin is a complex process mediated by thinning of the epidermis, loss of elastic tissue, and reduced collagen production. In addition, reduced activity and generation of cell types including keratinocytes, fibroblasts, and melanocytes results in the loss of tensile strength and elasticity of the skin [30]. Exosomes are thought to potentially mitigate and/or reverse these trends by their mediation of oxidative stress and inflammatory pathways [31]. Additionally, exosomes are believed to improve skin function by rejuvenating skin tissue through reduced expression of MMP and increased collagen and elastin production [32]. These results have been corroborated in several in vitro and animal studies.

In a study by Hu et al., they demonstrated that exosomes derived from human dermal fibroblasts caused increased procollagen type I expression and a significant decrease in MMP‐1 expression in both in vitro and in mice models [33]. A similar study by Oh et al. showed that exosomes derived from human induced pluripotent stem cells (iPSCs) protected cells from UVB damage and caused decreased levels of MMP‐1 and increased production of collagen [34]. Liang et al. [35] also showed the same trend of decreased MMP‐1 and MMP‐3 mRNA expression and increased collagen expression in rats treated with ADSC‐derived exosomes. In a clinical trial examining exosome use for skin rejuvenation, 56 patients applied topical exosomes from human platelet extract twice a day for 6 weeks. At the end of the 6‐week period, quantifiable imaging techniques of skin health demonstrated a significant reduction in redness, wrinkles, and melanin production and significant improvements in luminosity and color evenness [36]. Further clinical trials are needed to evaluate the full scope of efficacy of exosomes for facial rejuvenation and anti‐aging purposes.

A 31‐year‐old female receiving treatment for freckles and skin rejuvenation using exosomal therapy had two sessions of Picosure Pro using focused and zoom tips followed by exosome delivery with Turtle Pin 1.0 mm. The patient continued her topical treatment with non‐hydroquinone and retinol creams throughout. The before (left) and after (right) images of her results are provided in Figures 19, 20, 21, 22. The patient reported her satisfaction with the procedure to be a 10/10. The images show improvement in the pigmentation and general presentation of her skin.

### 5.1 Limitations

Limitations of exosomes include a lack of standardization in their production, preparation, delivery, and application between exosomes made by different manufacturers and/or derived from different tissue sources. This makes comparisons between the safety and efficacy of different exosome products exceedingly difficult. The current lack of FDA approval for exosome products and their derivatives, while studies are ongoing, has further contributed to the lack of regulation. Additionally, there are no medical guidelines or expert consensus recommendations on the appropriate use and indications for exosomes. There have been no major serious safety signals reported with the use of exosomes to date, but further studies are needed to fully describe the safety profile of exosomes for different indications and to monitor the long‐term impacts of their use. Comparator studies between widely accepted and proven therapies versus exosomes may also be of value to establish their value, or lack thereof, as a therapeutic option.

## 6 Conclusions

The initial evidence supports that exosomes may present an effective and novel therapeutic option that can potentially be considered in the treatment armamentarium of numerous aesthetic complaints including alopecia, scarring, hyperpigmentation, and facial rejuvenation. While further clinical data is required, the preliminary evidence is promising and suggests that exosomes may be considered as a treatment option for these conditions. As new sources and indications of exosomes are investigated, the therapeutic domain of exosomes will continue to expand. Additionally, the initial safety profile of exosomes has been strong and shows them to be well tolerated, but further investigation is necessary to further establish the full scope of their safety profile.

## Author Contributions

Milaan Shah, Victoria Dukharan, Luke Broughton, Carol Stegura, Luna Samman, Nina Schur, and Todd Schlesinger all contributed to the planning, review, synthesis, and writing of the manuscript. All authors have read and approved the final manuscript.

## Conflicts of Interest

Todd Schlesinger serves as a consultant, investigator, speaker, and/or advisor for Abbvie, Almirall, Allergan (An Abbvie company), ASLAN Pharma, Arcutis, Biofrontera, Beirsdorf, Benev, Bristol‐Myers Squibb, Castle Biosciences, Galderma, Eli Lilly, ExoCoBio, Incyte, Janssen, LEO, L'Oreal, Novartis, Pfizer, Regeneron, Sanofi, Sun Pharma, Takeda, UCB Pharma, and Verrica. The other authors declare no conflicts of interest.

## Data Availability

Data sharing not applicable to this article as no datasets were generated or analysed during the current study.
